# Supplementary material for: Understanding the leading indicators of hospital admissions from COVID-19 across successive waves in the UK
Source: Epidemiol Infect. 2023 Sep 4;151:e172. doi: 10.1017/S0950268823001449 (PMC10600913; doi:10.1017/S0950268823001449)
Supplement: Mellor et al. supplementary material [file S0950268823001449sup001.docx]

**Supplementary Information**

Supplementary Section A

We compare using a cross correlation analysis the national ONS positivity estimates over time with COVID admissions with a positive test within two days of arrival, to understand community infection. *Supplementary Figure 1* shows that there is a strong autocorrelation between positivity and admissions, greater than 0.8 for several days. However, while this relationship is leading, it peaks between 0 and 5 days, indicating a lead of less than one week. When taking into the data latency of over a week, this lead is no longer operationally actionable from a public health policy perspective.


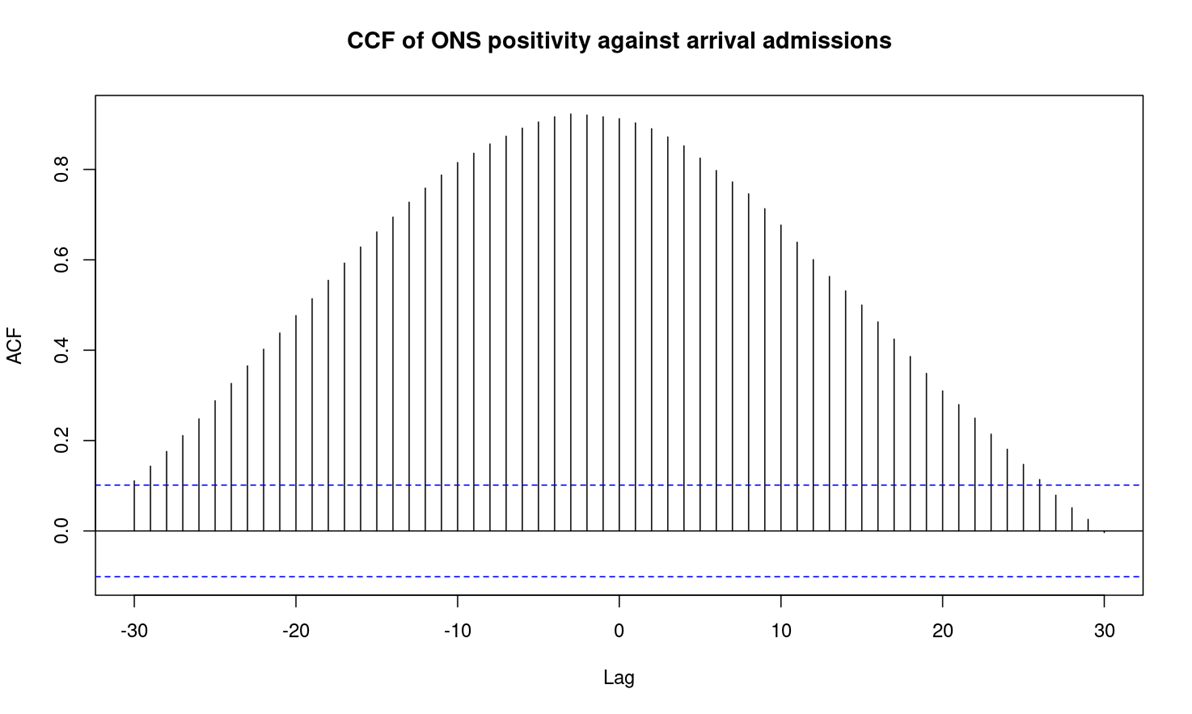


Supplementary Figure 1. Cross correlation analysis of ONS CIS national all age positivity against hospital admissions with a positive test within two days of arrival between 1^st^ October 2021 and 9^th^ October 2022. High ACF corresponds to strong correlation at a temporal offset between time series. The location of the peak indicates the corresponding best lag time (negative lead time)

| **Name** | **Start date** | **End date** |
| --- | --- | --- |
| BA.1 | 2021-11-15 | 2022-02-22 |
| BA.2 | 2022-02-23 | 2022-05-25 |
| BA.4/5 | 2022-05-26 | 2022-08-29 |

Supplementary Table 1. The start and end dates used to define each Omicron wave. The dates were chosen based on turning points in national trends, minimising the 7 day rolling average national hospital admission count.

Supplementary Section B

**Google Data Collection Logic**

The Google Trends data was curated within the UKHSA team that conducted this analysis, collected by API and processed. For this reason, how this data is processed is outlined below.

How Google Trends data is presented is described within the Google Trends FAQ [29]. For a given query of the API, or webpage, a metric based on a sample of recent/historic searches are returned, allowing for real time processing, rather than analysis of the whole Google Search data set.

The Google values for a given search term, location and point in time are not total search volume in that stratification. Instead, the values are normalised by total searches in the geography and time, giving a relative search volume comparable across areas at a given time. The resulting normalised values are scaled to within 0-100 based on how popular the term is compared to all other terms. This allows for useful comparison in relative search volumes across different regions (spatially normalised) and overall size of volume (scaled against other trends).

To collect this relative search volume data for a range of terms we go through the following steps utilising a modified version of the *pytrends* unofficial Google API python package [46] and post-download processing.

1. For a given search term “cough” - request the interest by region for “cough”, which gives a score for each city within the region of interest, per hour, recording the term, location, time and value. Store the relative search volume for a search term in a csv file.
2. Repeat the above steps each hour for the given search term, if the request fails repeat the request.
3. Once per day, load in all csv files for a given term, time, geography and append them into a single csv.
4. Once per day, across all terms, load all the daily csv files into memory.
5. Deduplicate through averaging any repeated terms spatially or temporally.
6. Using the coordinate of each geography provided by Google convert this to a nation, region, LTLA name and code. Convert areas within London to a single geography.
7. Find the mean of the hourly values per day for a given trend and low-level geography (LTLAs and London).

Supplementary Section C

**Table of variables**

| **Data Source** | **Grouped Variable** | **Description** | **Variables/Terms** |
| --- | --- | --- | --- |
| Google Trends | Google Entity | Collection of COVID symptom terms queried as entities in Google Trends rather than exact matching terms. | “Shortness of breath”, “Ageusia”, “Ansomia”, “Fever”, “Sore throat”, “Cough”, “Fatigue” |
|  | Google Symptom+Test | Terms relating to questions about symptoms and testing. | “coronavirus symptoms”, “coronavirus test”, “covid 19 test”, “covid positive”, “covid symptoms”, “covid test”, “get test covid”, “temp 38”, “test for coronavirus”, “test for covid” |
|  | Google General | Terms relating to generic COVID ideas. | “can I go out”, “corona”, “corona virus”, “coronavirus”, “covid”, “covid 19”, “covid rules”, “face mask”, “how to put on a face mask”, “isolated”, “rule of 6”, “self isolation” |
|  | Google Common | Search terms relating to common COVID symptoms. | “anosmia”, “change of taste”, “continuous cough”, “constant cough”, “cough” “dysgeusia”, “fatigue”, “fever”, “loss of taste”, “loss of taste and smell”, “loss of taste”, “no smell”, “no taste”, “persistent cough” |
|  | Google Rare | Search terms relating to rare COVID symptoms. | “congested”, “diarrhoea”, “headache”, “myalgia”, “neuropathy”, “rash”, “sore throat” |
|  | Google Severe | Search terms relating to severe COVID symptoms. | “breathing difficulties”, “dyspnea”, “out of breath”, “short of breath” |
| NHS 111 Pathways | NHS 111 Self-care AGE | Combined pathways where the outcome was for the enquirer to not seek medical attention but selfcare, stratified by age group. |  |
|  | NHS 111 Clinical AGE | Combined pathways where the outcome was for the enquirer to receive a clinical assessment, such as a test, within a specified period, stratified by age group. |  |
|  | NHS 111 Ambulance AGE | Pathways where the outcome was for an ambulance to be sent for the enquirer, stratified by age group. |  |
| ZOE App | ZOE Common | Recorded app user counts relating to common COVID symptoms. | “Persistent cough”, “fever”, “fatigue”, “sore throat”, “loss of smell”, “chills or shivers”, “altered smell” |
|  | ZOE Rare | Recorded app user counts relating to rare COVID symptoms. | "sore throat",  "diarrhoea",  "headache",  "unusual muscle pains",  "unusual joint pains",  "eye soreness",  "blisters on feet",  "rash",  "skin burning" |
|  | ZOE Severe | Recorded app user counts relating to severe COVID symptoms. | “shortness of breath”, "delirium”, "chest pain", "brain fog” |
|  | ZOE Irrelevant | Recorded app user counts relating to symptoms irrelevant to COVID. | “skipped meals”,  “hoarse voice”,  “abdominal pain”  “tongue surface”,  “mouth ulcers”,  “nausea”,  “dizzy light headed”,  “red welts on face or lips”,  “hair loss”,  “feeling down”,  “sneezing”,  “Earache",  “ear ringing”,  “swollen glands”,  “irregular heartbeat”,  “other symptoms” |
| LFDs | LFD Positive Count | Count of positive LFDs reported. |  |
|  | LFD Count | Count of total LFDs reported. |  |
|  | LFD Positivity Rate | Proportion of positive LFDs by total LFDs. | LFD Positive Count / LFD Count |
|  | LFD Positivity Per Capita | Proportion of positive LFDs per person in the area. | LFD Positive Count / Population size |
| NHS COVID App | NHS COVID-19 App Risky Contact Notification | Number of risking notifications received. |  |
|  | NHS COVID-19 App received postive test | Number of positive PCR tests entered into the app = total of positive distinct tests. |  |
|  | NHS COVID-19 App LFD positive and isolating | Number of users in isolation – positive LFD (assisted and self admin) test result. |  |

Supplementary Table 2. Groupings of underlying measurements that generate variables for evaluation. Terms that were not shown to vary over time or had consistently zero values are excluded from the Google Trends groupings.


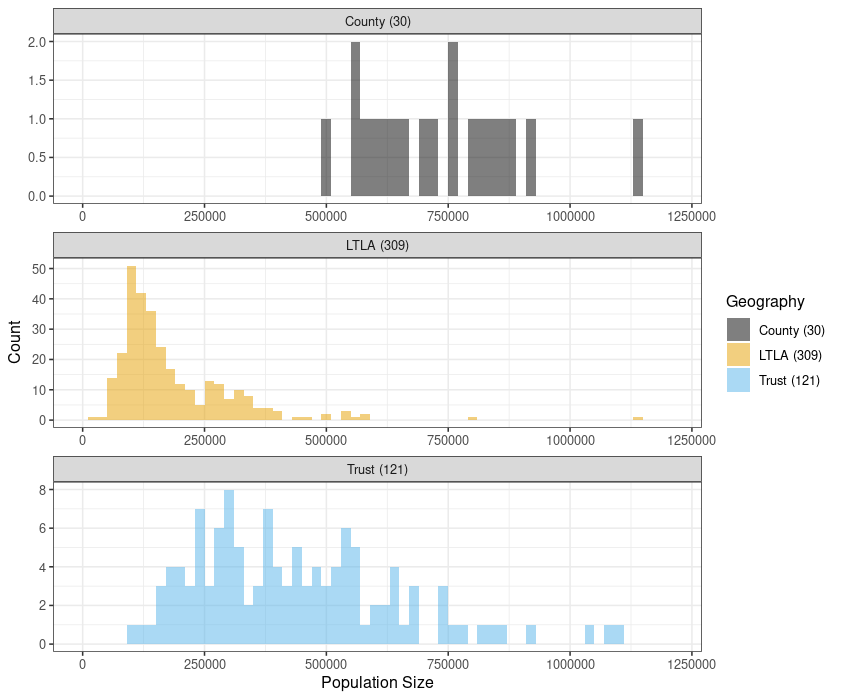


Supplementary Figure 2. The distribution of population sizes for NHS Trusts, LTLAs and administrative counties in England. The Trust populations are shown to be of comparable size to LTLAs and counties. Total counts are given in parenthesis. Counties are defined as area codes E10-E11. LTLAs are defined as area codes E06-9.

| **Data Source Name** | **End date** |
| --- | --- |
| Admissions | 2022-08-29 |
| Google Trends | 2022-08-01 |
| NHS 111 | 2022-08-29 |
| ZOE App | 2022-04-12 |
| LFD Tests | 2022-08-29 |
| NHS COVID App | 2022-09-29 |

Supplementary Table 3. The truncation date for each individual data source. ZOE data was truncated due to data sharing, and Google was truncated due to changes in the data source.


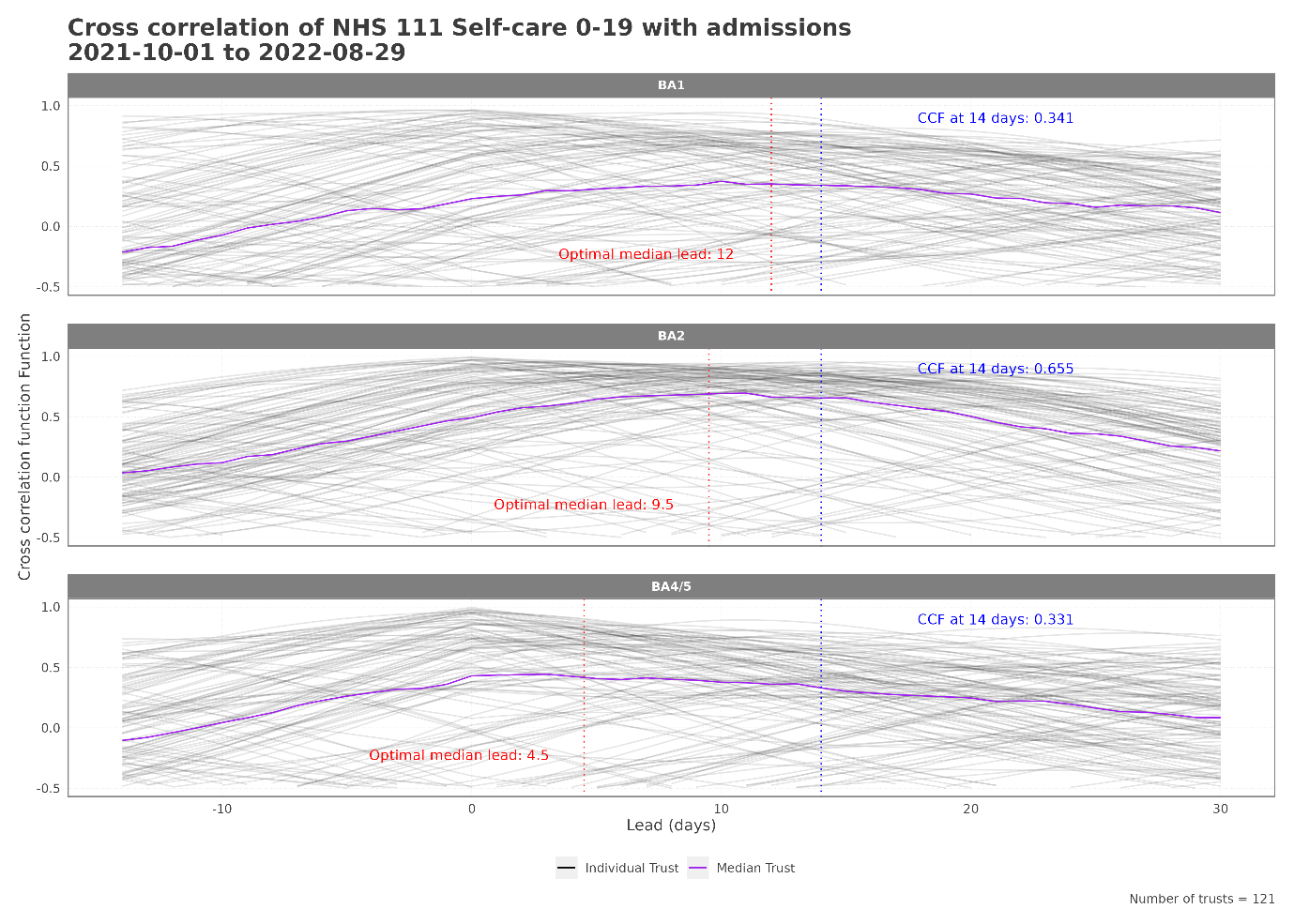


Supplementary Figure 3. Cross correlation function plot using NHS 111 selfcare treatments for 0–19-year-olds with key metrics highlighted for each wave. The optimal lead times are shown to highlight the difference in usefulness of an indicator across waves.


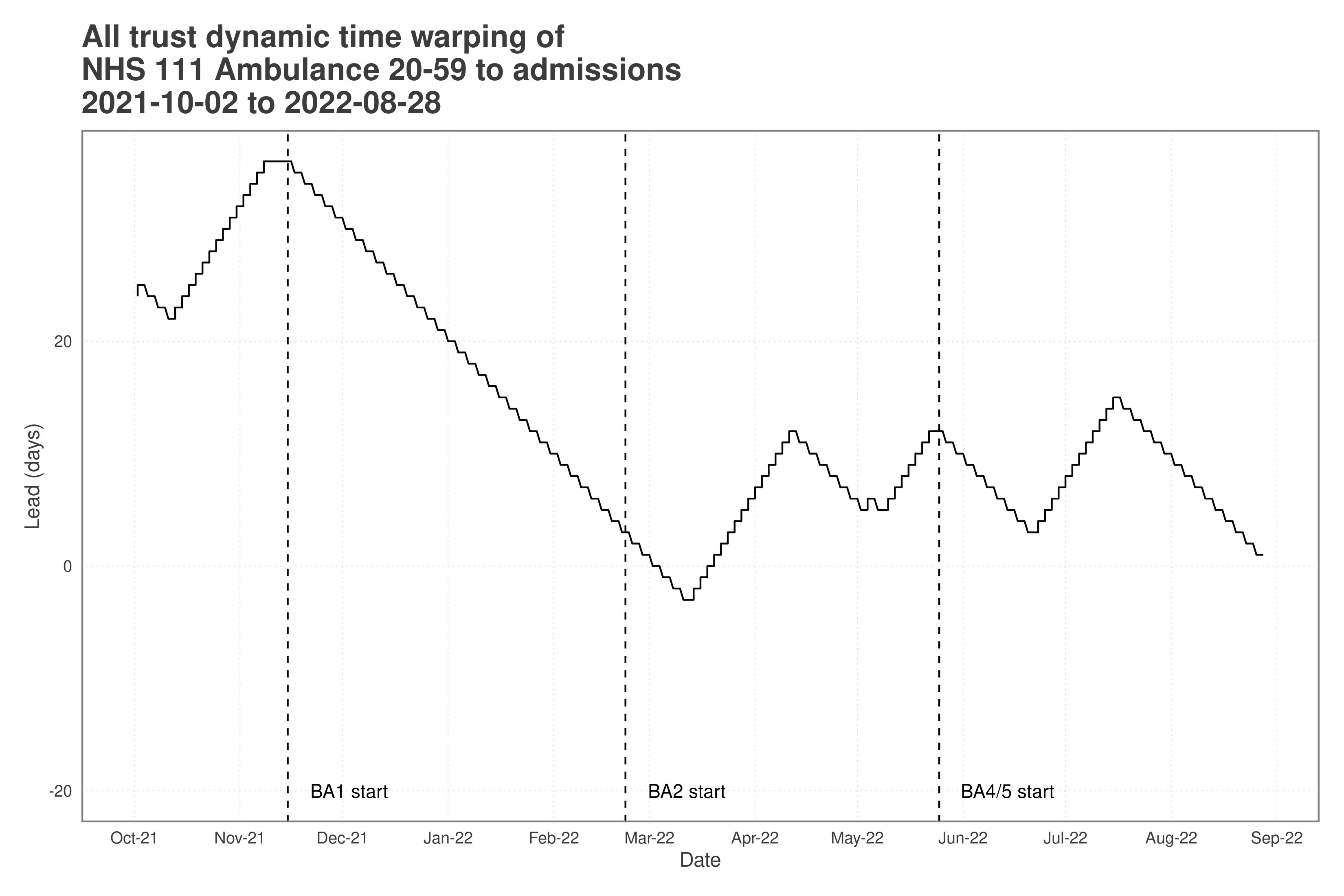


Supplementary Figure 4. The time shift between the two-time series variables at each point in time produced by the dynamic time warping alignment algorithm. The multivariate analysis allows the lead for the variable across all Trusts to be calculated.
